# Waist to height ratio; a simple and valid index for metabolic syndrome in Korean adolescents

**DOI:** 10.1186/1687-9856-2013-S1-P171

**Published:** 2013-10-03

**Authors:** In-Hyuk Chung, Sang Shin Park, Eun-Gyong Yoo

**Affiliations:** 1 Department of Pediatrics, College of Medicine, CHA University, Sungnam, Korea; 2 Department of Veterinary Integrative Biosciences, College of Veterinary Medicine and Biomedical Sciences, Texas A & M University, College Station, TX, USA

## 

The incidence of metabolic syndrome (MS) is increasing in adolescents, which can lead to major health threats in the future. Body mass index (BMI) and waist circumference (WC) are commonly used for identifying adolescents at higher risk for MS, but BMI is not a measure of fat distribution, and different BMI and WC cut offs are used according to age. Waist-to-height ratio (WHtR)can be a good indicator for MS, because it includes WC, a good proxy for visceral adiposity. Same WHtR cut off might be used throughout adolescence because it accounts for growth in height by age. We evaluated the validity of WHtR, when compared to BMI and WC, in identifying adolescents with MS.

We analyzed data for 4,068 adolescents aged 10-18 years from the Korean National Health and Nutrition Examination Surveys conducted between 1998 and 2008. MS was defined by International Diabetes Federation criteria. The receiver operating characteristic (ROC) curve was used to determine the ideal WHtR cut off. Area under the curve (AUC), sensitivity and specificity of WHtR for identifying MS were calculated from the ROC curve, and compared with those of BMI (≥ 95P for age and sex) and WC (≥ 90P for age and sex).

The prevalence of MS was 2.4% in boys, 2.1% in girls. The ideal WHtR cut off was 0.51 (sensitivity 94.3%, specificity 94.4%) for boys, and 0.48 (sensitivity 100%, specificity 87.6%) for girls. In ROC curve analysis, the AUC of BMI, WC and WHtR were 0.957, 0.971 and 0.966 in identifying MS for boys and 0.935, 0.965 and 0.961 for girls, respectively. For boys, the sensitivity of BMI, WC and WHtR (≥0.51)was65.4%, 100% and 98.1% and specificity was 95.5%, 89.6% and 89.3%, respectively. For girls, sensitivity of BMI, WC and WHtR (≥0.48) was 67.5%, 97.5% and 100% and specificity was 94.7%, 90.8% and 87.8%, respectively. WHtR is a simple and valid index for predicting MS in adolescents. WHtR is almost as useful as WC, and it has the advantage that age specific reference tables are not required.

